# Subcellular Clearance and Accumulation of Huntington Disease Protein: A Mini-Review

**DOI:** 10.3389/fnmol.2016.00027

**Published:** 2016-04-21

**Authors:** Ting Zhao, Yan Hong, Xiao-Jiang Li, Shi-Hua Li

**Affiliations:** ^1^Department of Human Genetics, Emory University School of MedicineAtlanta, GA, USA; ^2^State Key Laboratory of Molecular Developmental Biology, Institute of Genetics and Developmental Biology, Chinese Academy of SciencesBeijing, China

**Keywords:** huntingtin, neurodegeneration, proteasome, autophagy, aggregation

## Abstract

Huntington’s disease (HD) is an autosomal dominant, progressive neurodegenerative disease caused by an expanded polyglutamine (polyQ) tract in the N-terminal region of mutant huntingtin (mHtt). As a result, mHtt forms aggregates that are abundant in the nuclei and processes of neuronal cells. Although the roles of mHtt aggregates are still debated, the formation of aggregates points to deficient clearance of mHtt in brain cells. Since the accumulation of mHtt is a prerequisite for its neurotoxicity, exploring the mechanisms for mHtt accumulation and clearance would advance our understanding of HD pathogenesis and help us develop treatments for HD. We know that the ubiquitin-proteasome system (UPS) and autophagy play important roles in clearing mHtt; however, how mHtt preferentially accumulates in neuronal nuclei and processes remains unclear. Studying the clearance of mHtt in neuronal cells is a challenge because neurons are morphologically and functionally polarized, which means the turnover of mHtt may be distinct in different cellular compartments. In this review, we discuss our current knowledge about the clearance and accumulation of mHtt and strategies examining mHtt clearance and accumulation in different subcellular regions.

## Huntington’s Disease and Selective Neuropathology

Huntington’s disease (HD) is an autosomal dominant neurodegenerative disease that is characterized by motor abnormalities, cognitive decline and psychiatric problems (Munoz-Sanjuan and Bates, 2011). The disease is caused by the expansion of a trinucleotide CAG repeat in exon 1 of the HD gene, which encodes an expanded polyglutamine (polyQ) tract in the N-terminal region of mutant huntingtin (mHtt). While most HD patients carry CAG repeats in the range of 38–55 and develop neurological symptoms in mid-life, larger repeats (>60Q) can cause juvenile onset HD (Ross et al., 2014).

Despite the ubiquitous expression of mHtt in the brain and peripheral tissues, the major pathological feature of HD is selective neurodegeneration (Vonsattel and DiFiglia, 1998; Munoz-Sanjuan and Bates, 2011). Similarly, selective neurodegeneration is also seen in many other neurodegenerative diseases, such as Alzheimer’s and Parkinson’s diseases, suggesting that multiple factors may contribute to the selective neurodegeneration in these diseases. Given the known genetic mutation in HD and its well-characterized neuropathology, HD makes an ideal model for investigating how selective neuropathology occurs with aging. In HD, neuronal degeneration is characterized by the preferential loss of neuronal cells in the striatum in the early disease stage and extensive neurodegeneration in a variety of brain regions in later disease stages (Ross et al., 2014). This progressive neurodegeneration is consistent with the late-onset neurological symptoms of HD.

## Preferential Accumulation of mHtt in Neuronal Nuclei and Processes

The age-dependent neurodegeneration and neurological symptoms in HD correlate with the accumulation of misfolded forms of mHtt in neuronal cells. The expansion of the polyQ repeat in mHtt causes the misfolding of mHtt and formation of mHtt aggregates in neuronal nuclei and neuropils in HD patient brains (DiFiglia et al., 1997; Gutekunst et al., 1999). These aggregates are recognized by antibodies to the N-terminal region of mHtt, suggesting that the aggregates are formed by mHtt N-terminal fragments that are produced by the proteolysis of full-length mHtt. Indeed, transgenic mice expressing N-terminal mHtt show abundant mHtt aggregates in their neuronal nuclei and processes (Davies et al., 1997; Schilling et al., 1999). Studies of these transgenic HD mice also demonstrate that N-terminal mHtt fragments preferentially accumulate in neuronal cells to form nuclear aggregates and neuropil aggregates. HD knock-in mice express full-length mHtt at the endogenous level under the control of the mouse Htt gene, and therefore mimic HD patients genetically. In HD knock-in mice, mHtt also forms aggregates first in the neuronal nuclei of the striatum (Wheeler et al., 2000; Li et al., 2001; Lin et al., 2001). Although this preferential formation of neuronal nuclei aggregates mirrors the vulnerability of striatal neurons in HD patients, mHtt appears to form more neuropil aggregates than nuclear aggregates in HD patients in early disease stages (DiFiglia et al., 1997; Gutekunst et al., 1999). Despite this species-dependent difference, in mice expressing full-length mHtt and modeling early stages of HD, neuropil aggregates form preferentially in the lateral globus pallidus (LGP) and substantia nigra (SN; Li et al., 2001). The majority of striatal neurons extend their axons to the LGP and SN, two brain regions degenerated more significantly during the early stage of HD (Reiner et al., 1988; Richfield et al., 1995). Thus, the preferential accumulation of mHtt in both the neuropils and nuclei of striatal neurons may account for the selective striatal neurodegeneration in HD.

Many studies report that mHtt aggregates can be either toxic or protective. It is possible that mHtt aggregates are both harmful and beneficial depending on the disease stage, subcellular localization and their association with other partners or organelles. Nevertheless, because mHtt aggregates are formed by N-terminal mHtt fragments that are misfolded, the mHtt aggregates reflect the accumulation of misfolded mHtt. Growing evidence indicates that the misfolded mHtt exerts its neurotoxicity by disturbing a wide range of cellular functions (Ross et al., 2014). The wide range of cellular toxicity from mHtt is due perhaps to its ability to interact with a variety of proteins and to interrupt the function of these interactors (Li and Li, 2004; Shirasaki et al., 2012) via a gain-of-function mechanism. Thus, the accumulation of misfolded mHtt is a prerequisite to its neuronal toxicity, and the clearance of mHtt is key to the treatment of HD.

## Intracellular Clearance of mHtt

Two proteolytic machineries are critical for clearing misfolded proteins. One is the ubiquitin-proteasome system (UPS), which mainly clears soluble and short-lived proteins in eukaryotic cells. The other is autophagy, which removes long-lived proteins, aggregated proteins and damaged organelles.

Because the formation of mHtt aggregates is age-dependent, the initial notion was that mHtt impairs proteasomal function (Bence et al., 2001; Venkatraman et al., 2004). However, targeting expanded polyQ for proteasomal degradation did not compromise proteasome activity (Michalik and Van Broeckhoven, 2004). Also, mHtt-exon1, the shortest N-terminal fragment of mHtt, was completely digested by the proteasome (Juenemann et al., 2013). In addition, *in vivo* studies show that proteasome activity in HD mouse brains is not perturbed by mHtt expression (Wang et al., 2008a; Bett et al., 2009). Although no global impairment of proteasomal activity is seen, proteasomal dysfunction probably occurs in certain subcellular regions, such as axonal terminals, which may lead to the accumulation of mHtt in these places (Wang et al., 2008a). It is established that mHtt compromises the axonal transport of mitochondria (Orr et al., 2008; Reddy and Shirendeb, 2012). Since the UPS is a highly ATP-dependent system (Schrader et al., 2009), defective mitochondria transport may lead to ATP deficiency in neurites and nerve terminals, which can impair the local degradation of mHtt by the proteasome and causes the aggregation of mHtt in these subcellular regions.

Using various HD cell and animal models, previous studies have shown that upregulation of autophagy leads to a reduction of mHtt aggregates, indicating that autophagy plays a role in the clearance of mHtt aggregates (Qin et al., 2003; Sasazawa et al., 2015). K63-linked polyubiquitination on mHtt is proposed to confer the selectivity on the degradation of mHtt aggregates by autophagy since K63-ubiquitinated substrates are recognized by autophagy receptors, such as p62, which has been shown to bind Htt (Tan et al., 2008). However, Bhat et al. (2014) found that ubiquitination of K48 linkage on mHtt switches to K63 linkage with aging, which promotes aggregation. On the other hand, mHtt compromises autophagy by perturbing cargo recognition and autophagosome motility (Martinez-Vicente et al., 2010; Wong and Holzbaur, 2014). Since the ubiquitination of both K48 and K63 is involved in the clearance of misfolded proteins by the UPS and autophagy, how the UPS and autophagy work together to remove mHtt remains to be clarified. Histone deacetylase 6 (HDAC6) probably links autophagy to UPS for clearance of mHtt as autophagy is activated in an HDAC6-dependent manner when proteasomal function is impaired (Pandey et al., 2007).

## Strategies to Examine Subcellular mHtt Clearance and Accumulation

Given the fact that mHtt preferentially accumulates in neuronal nuclei and processes, it is important to determine mHtt clearance at the subcellular level. Nuclei and synaptosomes can be isolated via biochemical fractionation, and the fractions can be analyzed by Western blotting. The advantage of this assay is that aggregated and monomer proteins can be separated in SDS gel and assessed for their relative levels on the same blot. Quantifying the levels of soluble and aggregated mHtt can yield valuable information about the levels of mHtt in these subcellular regions; however, the combination of subcellular fractionation and western blotting cannot monitor the degradation of mHtt in real time. Moreover, it is difficult to separate the fraction that is enriched in neuronal processes or neuropil. In addition, fractionation requires the use of brain homogenates that cannot distinguish cell types in which mHtt may differentially accumulate or be cleared.

It is not feasible to study the degradation of mHtt in living neurons until phototransformable fluorescent proteins are invented. Phototransformable fluorescent proteins can be classified into three types-photoactivating, photoconverting and photoswitching-based on their responses to light. Currently, these proteins are used widely to study the dynamics of molecules and cells in a spatiotemporal manner (Adam et al., 2014). Tsvetkov et al. (2013) recently used Dendra2, one of the photoconvertible fluorescent proteins, to study the degradation of mHtt-exon1 in cultured striatal neurons. Dendra2 is a green-to-red photoconvertible fluorescent protein featuring fast maturation and bright fluorescence (Chudakov et al., 2007). Light irradiation at a 405-nm wavelength can efficiently activate Dendra2 and consequently switches its fluorescent color irreversibly from green to red. The linkage of Dendra2 to mHtt does not perturb its neurotoxicity or the property of aggregation in neurons. After transient transfection of Htt-exon1-Dendra2 into striatal neurons, Tsvetkov et al. (2013) converted green Htt-exon1-Dendra2 to red fluorescence by activating Dendra2 with 405-nm laser light. Therefore, after irradiation, the reduction of red Htt-exon1 over time reflects its degradation in live cells. Using this strategy, called the “optical pulse chase” assay, Tsvetkov et al. (2013) found that mHtt-exon1 was cleared faster than its wild-type counterpart in the body of the cultured striatal neurons.

Although optical pulse chase has been used mainly with *in vitro* cultured cells, it should also be useful for determining the turnover of mHtt in live cells at subcellular levels, such as neuronal processes and terminals. Examining the change of red fluorescence avoids the potential influence from newly synthesized proteins as they are labeled by inactive green Dendra2, so that the degradation of mHtt can be quantified. The Dentra-2 fusion proteins can be expressed via adenoviral-associated vectors (AAV), and stereotaxic injection would allow the delivery of the AAV vectors to specific brain regions, such as the striatum. The injected brain region can then be isolated and sectioned into brain slices, which can be observed in medium under a fluorescent microscope. Photoconversion of Dendra-2 will then be achieved to measure the clearance of mHtt in neuronal processes or nerve terminals in brain slices. Moreover, using different promoters would confer the expression of Dendra-2-mHtt in specific types of cells, making it possible to study the cell type-dependent degradation of mHtt in the brain. Further, tagging the Dendra-2 fusion protein with organelle targeting sequences would allow us to study mHtt’s degradation in specific organelles. Given that suppression of neuropil aggregates ameliorated the neurological symptoms of HD mice (Wang et al., 2008b), promoting clearance of mHtt by the UPS in neuropils should be therapeutically beneficial for HD. In support of this idea, overexpressing ube3a, an ubiquitin E3 ligase, can activate the UPS and decrease mHtt aggregates in the brains of HD knock-in mice (Bhat et al., 2014). In addition, up-regulating autophagy has also been found to eliminate mHtt aggregates. (Ravikumar et al., 2004; Sasazawa et al., 2015).

## Conclusion

Although mHtt is known to be cleared by the UPS and autophagy, how mHtt is cleared at the subcellular level remains unknown. Understanding this interesting issue will shed light on the pathogenesis of HD and also help us find ways of accelerating the clearance of this toxic protein. Biochemical fractionation would allow one to simultaneously examine the relative levels of aggregated and monomer mHtt on the same Western blot. The optical pulse chase assay would enable the study of the degradation of mHtt in different subcellular regions in living neurons. Such studies will answer the question of whether mHtt degradation is subcellular region dependent and could also be extended to studies of other types of misfolded proteins in different neurodegenerative diseases.

## Author Contributions

TZ, YH, X-JL and S-HL wrote this review article.

## Conflict of Interest Statement

The authors declare that the research was conducted in the absence of any commercial or financial relationships that could be construed as a potential conflict of interest.

## Abbreviations

AAV: Adenoviral-associated vectors
HD: Huntington’s disease
HDAC6: Histone deacetylase 6
LGP: Lateral globus pallidus
mHtt: Mutant huntingtin
polyQ: Polyglutamine
SN: Substantia nigra
UPS: Ubiquitin-proteasome system.
